# Does kinesio taping affect balance in individuals with multiple sclerosis?

**DOI:** 10.1007/s10072-025-08115-9

**Published:** 2025-03-25

**Authors:** Fatma Erdeo, Ali Ulvi Uca, Musa Çankaya, Neslihan Altuntaş Yılmaz

**Affiliations:** 1 Nezahat Keleşoğlu, Faculty of Health Sciences, Department of Physiotherapy and Rehabilitation, Necmettin Erbakan University, Konya, Turkey; 2 Necmettin Erbakan University, Faculty of Medicine, Konya, Turkey; 3 Seydişehir Health Services Vocational School Therapy and Rehabilitation Department, Necmettin Erbakan University, Konya, Turkey

**Keywords:** Kinesio taping, Standing balance, Multiple sclerosis, Rehabilitation

## Abstract

**Background:**

Balance disorders are common in individuals with multiple sclerosis (MS) due to the combined effects of lack of adequate postural control, muscle weakness, ataxia, and lack of sensory information. The aim of this study was to assess the effect of Kinesio taping (KT) on balance among subjects with MS.

**Method:**

This was a non-controlled observational study. A consecutive convenience sample of 30 individuals with MS was assessed. KT was applied directly to the skin under the foot. Clinical assessments were performed at baseline, immediately before and after application of the tape. Balance was evaluated with both eyes open and closed. The effects of sense of balance, spasticity, and muscle strength were evaluated.

**Results:**

A significant difference was observed before and after banding in the dominant and non-dominant extremities (p_before_ = .001, p_after_ = .002). There was a significant difference between vibration and two-point discrimination and balance (p > 0.05). There was no significant relationship between light touch sense and balance (p > 0.05). Further, except for tibialis anterior muscle strength, there was no significant difference between balance and other lower extremity muscles (p > 0.05). In addition, except for muscle spasticity in the quadriceps, there was a significant difference between balance and other lower extremity muscles (p < 0.05).

**Conclusions:**

KT appears to be a useful tool in reducing the fall rate and improving balance skills in patients with MS. These preliminary results suggest that the use of Kinesio foot taping may be useful in immediately stabilising body posture.

## Introduction

Multiple Sclerosis (MS), which affects the brain, brain stem, spinal cord, and optic nerves, causes a wide variety of symptoms that can affect balance and gait [1]. These include weakness, spasticity, and fatigue, as well as changes in sensation, coordination, vision, cognition, and bladder function. The majority of individuals with MS exhibit postural control and gait abnormalities even in the early stages of the disease [2]. The reason for the frequent occurrence of balance disorder in individuals with MS is probably due to the combined effects of lack of adequate postural control strategies, weakness, ataxia, and lack of reliable sensory information. [3]

Correct perception of physical stimuli by the sensory receptors of the visual, somatosensory and vestibular systems and the integration of these inputs are necessary for a good posture balance [4]. Disturbances in the sensory system may cause inadequate motor responses, which are often observed in individuals with MS. Previous research has shown that individuals with MS have shorter stance times in a narrow area of support than healthy individuals. [5]

Kinesiologic taping (KT) is a therapeutic modality used in the treatment and management of various conditions that cause musculoskeletal and neuromuscular deficits.Previous studies have shown that KT has been used to relieve pain, correct joint malalignment, reduce swelling, provide support to muscles, and increase or inhibit muscle recruitment [29]. KT stimulates skin mechanoreceptors, improves local circulation, and modulates pain [31]. Previous studies to determine the effects of KT on receptors have reported that the use of KT on specific muscles and joints can increase muscle excitability [31]. Studies have reported that taping applied over the skin stimulates cutaneous mechanoreceptors, allowing more sensory signals to be carried to the central nervous system for integration in any deformity.By providing continuous afferent stimulation through the skin, KT improves proprioception and allows correction of improper position. Kinesio Taping (KT) is used to normalize balance and muscle tone. [30]

However, the number of studies showing its effectiveness in individuals with MS is limited. The aim of this study is to evaluate the effect of KT applied to the soles of the feet on eyes open and closed balance in individuals with MS.

## Method

### Participants

The G*Power 3.1.9.2 program was used to calculate the sample size. Sebastião, et al., (2024) in their study on the effect of kinesiology taping on static balance in patients with MS, found that the effect size of the results of overt static balance was 0.60 [29]. This finding was used to calculate the sample size in our study. Using the G*Power 3.1.9.2 program with an effect size of 0.6, a standard error of 0.05, and a power of 95%, it was determined that the calculation made in the form of t tests; Correlation: Point biserial model should be performed with 27 participants. It was predicted that there might be %20 losses during the study, and 33 patients were included in our study [30]. Three participants were excluded from the study because one of them could not perform the tests in the study, one participant was not native Turkish reading and comprehension ability, and one participant did not want to participate in the study.

The diagnosis was determined by considering the 2010 Revised McDonald Criteria [28]. In addition, differential diagnosis includes cerebrospinal fluid examinations with lumbar puncture and magnetic resonance imaging. [27] The diagnosis was made by a physician with more than 20 years of experience. Thirty patients diagnosed with MS were included in the study. Expanded Disability Status Scale (EDSS) was kept between 1–4.5 (3.92 ± 0.69) because excessive spasticity and weakness may affect balance in more severely disabled patients. Inclusion criteria were as follows: at least 2 years since diagnosis, no recurrence within 4 weeks, no orthopedic disability, and no allergy to the tape.

The mean age of MS patients, consisting of 7 males and 23 females, was 45.03 ± 9.46 years. All individuals with MS who came to the clinic agreed to participate in the study and signed an informed consent form. First, the patient's balance without KT was evaluated with eyes open (EO) and closed (EC). 15 min. After resting, KT was applied to the sole of the foot.

**Fig. 1:**
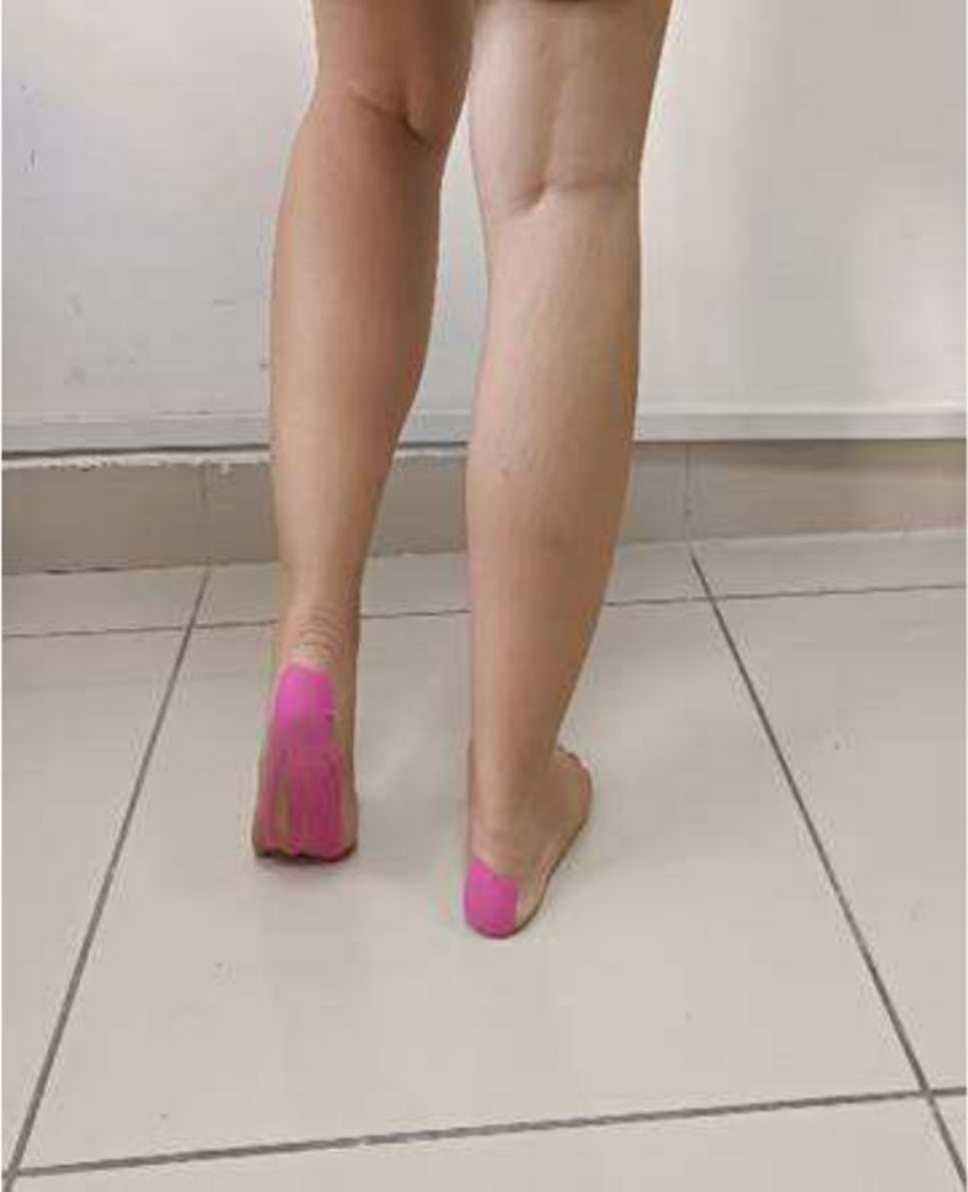
KT application method

In the supine position, 5 × 10 cm KT was applied starting from the end point of the Achilles tendon in the heel region and continuing to the metatarsal heads. Starting from a single piece at the heel, the application was divided into 4 pieces parallel to the fingers when it reached the metatarsal heads. The correction I technique (mechanical correction) was applied with 50% tension in 1/3 of the heel area (Fig. 1) [32, 33].

Balance with (EO) and (EC) was evaluated again. At the beginning of the study, light touch, discriminative touch, vibration, spasticity, and lower extremity muscle strength were evaluated. This study was approved by Necmettin Erbakan University, Health Sciences Ethics Committee (Decision Number: 2023/392). and performed in accordance with the Helsinki Declaration.

### Instrumental assessment

1. **One-leg stance test (SLST):** During the one-leg stance test, the patient was asked to lift one foot with eyes open so that it would not touch the supporting leg. The maximum test time is 30 s. The individual was asked to maintain this position. If the lifted leg touches the support leg, if the foot touches the floor, if there is a bounce or jump, or if any object in the environment is touched for support, the test was terminated considering that there was a balance disorder. [6]
2. **Light Touch Sense:** Semmes–Weinstein monofilaments (Smith & Nephew, Inc, Germantown, WI) were used to determine foot sensitivity. This test is known for its validity, reliability, reproducibility, and responsiveness and is widely used in research as well as in clinical settings [7]. Each patient was evaluated with 3.61 (0.4 mg) (decreased light touch sensation) in a long sitting position.
3. **Two-Point Discrimination:** The two-point discrimination (TPD) threshold value is expressed as the minimum distance at which two mechanical stimuli applied simultaneously to the skin can be perceived as two separate points. Due to the ease of application and interpretation of the TPD test, this evaluation method is frequently used in clinical and scientific studies [8]. The sole of the foot was evaluated from three locations: front, middle and hind.
4. **Vibration:** A 128 Hz tuning fork was used to evaluate the sense of vibration. For control purposes, it was first applied to different parts of the body to make the patient familiar with the test. The tuning fork was first vibrated by striking a hard surface and then the forked end was touched to the medial malleolus of both the right and left feet. The average value of the three tests was taken into consideration. [9]
5. **Muscle Test:** Using a handheld dynamometer (HHD), the gluteus maximus and hamstrings were evaluated in prone position and knee flexion, hip flexion, knee extension, ankle dorsi, and plantar flexion were evaluated in sitting position. [10]
6. **Spastisity Assesment:** The Modified Ashworth Scale (MAS) is the most common spasticity assessment used. It is the most common assessment used by therapists in clinical and research studies [11]. Hip flexion was evaluated in the supine position, and gastrocnemius spasticity was evaluated in the long sitting position.

### Statistical analysis

Statistical analysis was performed using the SPSS software package for Windows (version 29; SPSS, IBM Cooperation). Data are presented as mean ± standard deviation. The normality of the data distribution was evaluated using the Shapiro–Wilk test. Further, the Wilcoxon Ordered Signs Test was used for data that did not have a normal distribution [12]. Cohen’s d effect size’was also studied. The effect size was defined as small (0.2–0.49), medium (0.5–0.79), and large (|< 0.8). Further, Pearson distribution analysis and Spearman distribution analysis were employed to evaluate the relationships among quantitative variables. In addition, p < 0.05 was considered statistically significant.

## Results

30 individuals with MS were included in the study. Demographic characteristics of the participants are shown below (Table 1).

**Table 1:** Distribution of descriptive characteristics (*N*: 30)

Variables	Multiple Sclerosis (N: 30)	
	$\overline{x }$ ± SS	
Age	45.03 ± 9.46	
EDDS	3.92 ± 0.69	
	**N**	_**%**_
Gender		
Man	7	23.3
Woman	23	76.7

Muscle strength averages and the difference between right and left are shown in Table 2.

**Table 2:** Dominant and non-dominant extremity muscle strength

Variables	Dominant Ekstremity X ± S	Nondominant Ekstremity X ± S	z	p
Hip Flexor M	27.85 ± 8.58	25.50 ± 6.80	−2.82	0.005
Knee Extansor M	30.75 ± 8.32	26.99 ± 8.25	−3.15	0.002
Gluteus Max. M	22.59 ± 5.69	21.70 ± 5.79	−1.26	0.206
Tibialis Antreior M	20.21 ± 7.53	20.95 ± 8.07	−0.730	0.465
Gastrocnemius M	22.39 ± 6.98	28.85 ± 37.40	−0.298	0.765

*X* Ortalama, *S* Standart Sapma, Wilcoxon İşaretli Sıralar Testi

Considering the effect of KT on eo and ec balance, no effect of KT on balance (ec) was found (p > 0.05). (Table 3).

**Table 3:** SLST Results of the study group without KT and with KT

	Without KT X ± SS	With KT X ± SS	es	p
SLST(Dominant extremity)(eo)	9.63 ± 10.19	10.86 ± 10.98	0.11	**0.000**
SLST(Nondominant extremity)(eo)	8.16 ± 9.72	12.76 ± 11.30	0.43	**0.002**
SLST(Dominant extremity)(ec)	3.63 ± 6.60	3.87 ± 6.58	0.03	0.186
SLST(Nondominant extremity)(ec)	2.96 ± 5.79	2.94 ± 4.28	0.39	0.245

*SLST *Single Leg Stance Test, *KT *Kinesiotape, *eo *eyes open, *ec *eyes close, *es *effect size, ꞎ Wilcoxon İşaret Testi *p* < 0.05

As a result of the correlation between muscle strength and balance, a significant difference was found between dominant extremity tibialis anterior muscle strength and balance (p < 0.05).

The correlation of spasticity and base senses with balance is shown in Tables 4 and 5.

**Table 4:** Correlation of spasticity with SLST

Spastisity	Without KT (eo) dominant rp		Without KT (eo) nondominant rp		Without KT (ec) dominant r p		Without KT (ec) nondominant rp		With KT (eo) dominant rp		With KT (eo) nondominant rp		With KT (ec) dominant rp		With KT (ec) nondominant rp	
Hip Flexor muscle (dominant foot)	−0.292	0.117	.−216	0.252	−0.270	0.149	−0.038	−0.840	−0.210	0.265	−0.236	0.218	−0.406*	**0.026**	−0.275	0.141
Hip Flexor muscle (non-dominant foot)	−0.559**	**0.001**	−0.338	**0.068**	−0.457*	0.011	−0.233	0.216	−0.523**	**0.003**	−0.449*	**0.015**	−0.503**	**0.005**	−0.434*	**0.016**
Quadriceps (dominant)	0.073	0.702	0.083	0.662	0.141	0.459	0.350	0.058	0.119	0.530	0.127	0.513	−0.175	0.354	−0.027	0.170
Quadriceps (nondominant foot	0.374*	**0.042**	−0.104	0.585	−0.342	,064	0.063	0.739	−0.316	0.089	−0.211	0.272	−0.376*	**0.041**	−0.257	0.170
Gastroknemius (dominant)	−0.227	0.228	−0.051	0.787	−0.393*	0.0**32**	−0.093	0.625	−0.199	0.344	−0.182	0.344	−0.351	0.058	−0.197	0.297
Gastroknemius (non-dominant)	−0.233	0.236	−0.096	0.614	−0.373*	**0.043**	−0.290	0.120	−0.261	0.164	−0.340	0.071	−0.244	0.194	−0.233	0.216

**Table 5:** SLST correlation with base senses

Variables	Without KT (eo) dominant rp		Without KT (eo) nondominant rp		Without KT (ec) dominant r p		Without KT (ec) nondominant rp		With KT (eo) dominant rp		With KT (eo) nondominant rp		With KT (ec) dominant		With KT (ec) nondominant	
Vibration (dominant foot)	0.441*	**0.015**	0.407*	**0.026**	0.365*	**0.048**	0.314	0.091	0.438*	**0.016**	0.491**	**0.066**	0.511**	**0.004**	0.508**	**0.004**
Vibration (non-dominant foot)	0.441	**0.015**	0.425*	**0.019**	0.330	0.075	0.326	0.079	0.415*	**0.023**	0.417*	**0.022**	0.510**	**0.004**	0.455*	**0.012**
Fore foot Discrimination (dominant)	−0.528**	**0.003**	−0.319	0.086	−0.415	0.023	−0.161	0.395	−0.615**	**0.000**	−0.546*	0.002	−0.374*	**0.042**	−0.380*	0.0**38**
Fore foot Discrimination (non-dominant)	−0.211	0.264	−0.024	0.898	−0.171	0.365	−0.102	0.594	−0.229	0.433	−0.149	0.440	−0.214	0.257	−0.098	0.607
Mid Foot Discrimination (dominant	−0.194	0.306	**-**0.186	0.326	−0.068	0.723	−0.263	0.160	−0.149	0.433	−0.249	0.433	−0.249	0.192	−0.243	0.196
Mid Foot Discrimination (nondominant)	−0.250	0.182	−0.308	0.098	−0.104	0.586	−0.218	0.247	−0.292	0.117	−0.359	0.056	−0.206	0.274	−0.310	0.095
Hind Foot Discrimination (dominant)	−0.266	0.156	−0.328	0.077	−0.166	0.381	−0.388*	**0.034**	−0.277	0.138	−0.359	0.056	−0.347	0.060	−0.342	0.064
Hind Foot Discrimination (nondominant)	−0.372*	**0.043**	−0.297	0.112	−0.217	0.249	−0.306	0.100	−0.349	0.058	−0.396*	**0.033**	−0.396*	**0.011**	−0.388*	**0.034**
Medial of fore foot (Semmes–Weinstein M.) (dominant)	0.411*	**0.024**	0.425*	**0.019**	0.513**	**0.004**	0.326	0.079	0.404*	**0.023**	0.417*	**0.027**	0.553**	**0.002**	0.455*	**0.012**

Significant correlations were found between vibration and forefoot light touch sense and balance (*p* < 0.05). There was no significant correlation between the lateral forefoot, midfoot, and lateral and medial hindfoot (*p* > 0.05). In two-point discrimination, eo and ec of the dominant forefoot were correlated with balance (*p* < 0.05)

## Discussion

In this study, we investigated the effect of KT applied to the sole of the foot using EO and EC SLST. We also examined the sensation, muscle strength, and spasticity that affect the duration of the SLST [13]. Determined that the SLST is one of the most appropriate clinical tests to evaluate balance in MS patients [14]. Therefore, we used SLST in our study. In previous studies, balance on two legs and oscillations in the antero-posterior and mediolateral directions were examined [14, 15]. The primary outcome of this study revealed that KT can improve balance on one leg with EO. However, no difference was observed when the eyes were closed. In the study by Cortesi et al. (2011), KT was applied and balance was evaluated with a stabilometer. It was observed that KT reduced oscillations in the antero-postero direction, but had no effect on oscillations in the mediolateral direction [16, 17]. In addition, extant studies have reported that the mechanical effect of taping on the foot and calf skin can increase the pressure on skin receptors, thereby improving kinaesthetic and joint position sense. [18] However, the effect of this result on balance has not been mentioned. When the parameters affecting the balance are examined, the literature reveals different results [19]. In the study conducted by Çitaker et al., seven muscles were evaluated and it was found that these muscles were associated with the balance of standing on one leg [20]. In the study by Soyuer and Mirza, muscles were evaluated manually and a weak correlation was revealed between lower extremity muscle strength and SLST [21]. In our study, a significant difference was observed only between tibialis anterior muscle strength and EO and EC SLST (p < 0.05). Further, evidence that spasticity is associated with gait and balance is limited [10, 22]. Gastro-soleus spasticity was evaluated in the study of Sosnoff et al. They showed that gastro-soleus spasticity is associated with mobility and balance. [23] In our study, hip flexors, quadriceps, and gastrocnemius muscles were evaluated. While hip flexor spasticity decreased in the dominant extremity, balance was significantly increased. In the non-dominant extremity, a significant correlation was observed in balance with EO without KT, while significant improvements were observed in balance with EO and EC with K.

In order to gain appropriate muscle activation to achieve movements such as walking and balance, the integration of sensory input with the motor plans and the central nervous system should be provided. The present results confirm previous findings that sensation is associated with balance and mobility outcomes in MS [24]. In order for the rehabilitation process to be carried out successfully, it is important to know which sense independently affects the balance ability. Park et al. In their study, it was stated that vibration was a strong influencer for the Functional Reach Test [25]. Jamali et al. He evaluated ataxia along with spasticity in his study, but since the Time up and go test and functional reach test were used in the study, no information was given about the state of balance with eyes closed [26]. In our study, since the balance on one leg with eyes closed was evaluated, information about the effectiveness of the senses could also be obtained. It was found that the sense of vibration eo and ec were effective in both cases, and the sense of light touch was more significant in the medial of the forefoot with eyes closed.

## Limitation

The most important limitation of the current research is that spasticity is evaluated at rest, not during walking or balance tasks. Because reflexes are dependent on limb position and task, future research should evaluate spasticity during mobility and balance tasks. In addition, the presence of a control group will provide a more objective evaluation of the results.

## Conclusion

KT application affects the balance with eyes open in individuals with MS. However, the lack of visual input affects the balance even though it is KT. In individuals with MS, balance on one leg affects more than sensory problems, spasticity and muscle strength. In our study, muscle strength of hip flexor, extensor, quadriceps, gastrocnemius, and tibialis anterior muscles was evaluated. In future studies, besides all lower extremity muscles such as trunk muscles and lower extremity inverters and evertors, parameters such as ataxia and endurance can be examined. Declaration of interest We thank MS patients for their participation in the study.

## Data Availability

The datasets generated during and/or analyzed during the current study are not publicly available, but are available from the corresponding author on reasonable request.
